# Reflexive thematic analysis of emergency department medical records of dementia patients regarding the identification of last days of life

**DOI:** 10.1017/S1478951525100527

**Published:** 2025-10-06

**Authors:** Sara Vieira Silva, Carla Teixeira, Bárbara Antunes

**Affiliations:** 1 Palliative Care Service, Centro Hospitalar Universitário de Santo António, Porto, Portugal; 2 Faculdade de Medicina da Universidade do Porto, Universidade do Porto, Porto, Portugal; 3 Intensive Care Unit, Centro Hospitalar Universitario de Santo Antonio, Porto, Portugal; 4 University of Porto Institute of Biomedical Sciences Abel Salazar, Porto, Portugal; 5 Department of Public Health and Primary Care, University of Cambridge, Cambridge, UK

**Keywords:** Medical records, Emergency department, Dementia, End-of-life, Reflexive thematic analysis

## Abstract

**Objectives:**

End-of-life care in the Emergency Department (ED) can be a challenge. Defining goals of care in dementia patients may be more complex. The quality of ED medical records is relevant for better care in the last hours or days of life. In this article, we explore the identification of last days of life recognition in ED records of dementia patients.

**Methods:**

Retrospective qualitative review of ED medical records of patients with dementia in the last 7 days of life using reflexive thematic analysis. This study was conducted at a university tertiary hospital, with a 24 h/7 days polyvalent ED. All 2021 ED medical records of dementia patients who presented to the ED within the last 7 days of their lives were included.

**Results:**

More than 1 in 4 patient’s medical records (n = 55, 27,4%) made no explicit reference to the identification of last days of life and only 2 medical records contained this specific designation. Most relevant issues presented under three broader themes: (I) diagnosis and prognosis concerning the last days or hours of life; (II) goals of care, medical decisions and communication about care in the last days or hours of life; and (III) comfort and needs assessment in the last days of life of patients with dementia in the ED.

**Significance of results:**

There is limited identification of the last days or hours of life in ED medical records and clinical notes are of poor-quality regarding communication and shared decision making.

## Introduction

The nature of the Emergency Department (ED) is to respond to very diverse acute and life-threatening conditions. Its response frequently includes rapid decision making and aggressive disease modifying therapy (Lafond et al. 2016). Patients with dementia often present to the ED, specially near the end-of-life (Vieira Silva et al. 2025). Appropriate end-of-life care in dementia has been proposed (van der Steen et al. 2014). It is recommended that care be person-centred, with communication and shared decision-making. It is essential to define goals of care and plan future interventions, to avoid overly aggressive, costly or non-proportional treatments, to provide optimal symptom management and comfort, with family care and involvement. Providing quality end-of-life care in the ED is likely to be a challenge. Although early preparation of Advance Care Planning is recommended, most patients with dementia don’t engage in these discussions early on(Donnelly et al. 2019; Karnieli-Miller et al. 2012; Yates et al. 2021). Cognitive impairment imposes greater complexity in defining goals of care (van der Steen et al. 2014), and, in fact, most patients with advanced dementia have significant limitations in their decision-making ability. As recommended, prioritizing of explicit global care goals helps guide care and evaluate its appropriateness. Advance Care Planning should start soon, when the patient can still be actively involved and patient preferences, values, needs and beliefs can be elicited. This plan should be regularly updated (Anantapong and Davies 2021) and the diminished capacity for a specific decision should not be assumed to compromise the person’s ability in other decisions(Hegde and Ellajosyula 2016). In advanced dementia and when death approaches, the patient’s best interest may be safeguarded with the primary goal of maximization of comfort and shared decision-making should involve the surrogate or family decision-makers. (van der Steen et al. 2014).

Failure to properly record patient preferences and decisions may lead to medical care incongruence, with patient’s wishes, as well as stress for patients and surrogate decision makers (Detering et al. 2010; Houben et al. 2014). Previous interventions to improve advance care planning documentation led to a significant increase in accessible electronic medical records (Kantor et al. 2021; Turley et al. 2016). However, Medical records (MR) in the ED are often of poor quality regarding patient preferences and decisions, which is a setting where professionals may have a greater need for access to this type information (Mashoufi et al. 2019, 2023; Sulmasy et al. 1996). Advanced care planning, as a continuous process of patient preferences, can help define the therapeutic plan and increase congruence with the patient’s wishes(Brinkman-Stoppelenburg et al. 2014). Medical records in the ED can benefit from this information but are not limited to it. They correspond to the medical notes, which can also include medical proposals of benefit to the patient, multidisciplinary and family discussion and decision-making in addition to documentation of clinical and comfort assessment(Brinkman-Stoppelenburg et al. 2014; Marck et al. 2014; Yash Pal et al. 2017). The MR in the ED is not limited to the aspects considered in the advance care planning, when this is available, but considers other aspects that may also be relevant to the quality of end-of-life care in this context. Therefore, good quality MR notes in the ED is important not only for patient care, but also to the efficiency and effectiveness of healthcare professionals and services(Marck et al. 2014; Yash Pal et al. 2017).

The present study aims to explore the quality of clinical notes regarding level of identification of the last days of life of patients with dementia in the ED MR. More specifically, our objectives are to identify aspects of communication to other healthcare professionals, patients and families, and measures of end-of-life care implementation.

## Methods

### Study design

We conducted a retrospective qualitative review of ED MR to explore clinical notes regarding the last days of life of patients with dementia who died in the hospital. The six steps approach proposed by Braun & Clarke (Braun and Clarke 2006) were followed: familiarisation with the data, generating initial codes, searching for themes, reviewing themes, defining and naming themes and producing the report.

Ethics approval [reference number 2023.094(083-DEFI/075-CE)] was provided by Unidade Local de Saúde de Santo António Ethics Commission and Review Board.

### Setting

Our study was conducted at a university tertiary hospital in Porto, Portugal, which serves a population of 650000 inhabitants with a polyvalent emergency service, 24 hours a day, 7 days a week.

### Participants

Patients with an established diagnosis of Alzheimer’s disease or other related dementias (ICD10 – F00* to F03) who died between January 1 and December 31, 2021 were eligible. Patients who resourced to the ED in the last 7 days of life were included. A total of 359 dementia patients died in hospital during this period. Of these, 201 patients presented to the ED in their last 7 days of life. We used descriptive statistical measures to explore quantitative clinical and service use details.

### Data generation

All electronic MR in the ED were approached chronologically from date of death to the last 7 days of life. We reviewed patient’s MR, including the entire period of hospitalization in the ED and the records of the different doctors, from different specialties, who followed the patient during that period. Descriptive summaries and de-identified extracts relevant to the aim and objectives were collected and extracted by one author (VSS), with verification by authors TC and AB (VSS researcher; TC clinical and academic professor of emergency and intensive care and AB academic researcher of palliative care). Quotes were extracted *verbatim*, translated into English and are presented in italics.

### Data analysis

Reflexive thematic analysis was used to qualitatively explore key issues and events surrounding ED-based end-of-life care for people with dementia (Braun and Clarke 2006, 2024). This methodology, considering our clinical and research experience, allowed us to better explore the subjectivity of the records. In line with existing literature, we inductively engaged with the following questions as a starting point for the analysis: Do the physicians identify the last days of life? How do physicians communicate to other healthcare professionals, patients and families about last days of life and related issues and do they record that in the patient MR? Which measures of care – in the last days of life – are recorded by physicians?

Analysis was conducted manually by two researchers (VSS and MP) who collaboratively reviewed codes and revised the themes, detailing inductive descriptive codes by marking similar phrases or words from the professionals’ narratives. Consensus coding was developed with both researchers coding the same transcripts and comparing them during regular meetings. Manual coding was chosen to facilitate familiarizing ourselves with details that automated tools may overlook. Themes were then generated by their shared meaning, around a central concept, while subthemes were considered as essential facets of these themes. Differences in coding and development of themes were further analysed with the wider research team until consensus was reached.

We adhere to the Reflexive Thematic Analysis Reporting Guidelines (Braun and Clarke 2024).

## Results

More than 1 in 4 patient’s MR (n = 55, 27,4%) had no explicit reference to the identification of the last days of life. Only 2 medical records contained this specific designation, specifically *“terminal”* and *“end of life.”*

During 2021, 359 patients with dementia who resourced to the ED, died in hospital. Of these, 201 patients (56,0%) were admitted to the ED in the last 7 days of life. The median age was 86,0 +-8,1 year and the majority of patients were female (58,7%, n = 118) (see Table 1), 94.0% (n = 189) with moderately severe/severe dementia (Functional Assessment Staging Tool ≥ 6). Almost half (49,8%, n = 100) presented at least one comorbidity, 79 (39,3%) had organ insufficiency, 16 (8,0%) malignancy and 4 (2,0%) had both. Most patients (n = 138) had two or more ED visits in the final 12 months of life, mean of 3,1 visits in that period. Most (n = 129) of the patients’ last ED visits occurred 3 to 4 days before their death, mean of 3,7 days.

### Key themes

**Figure 1.**
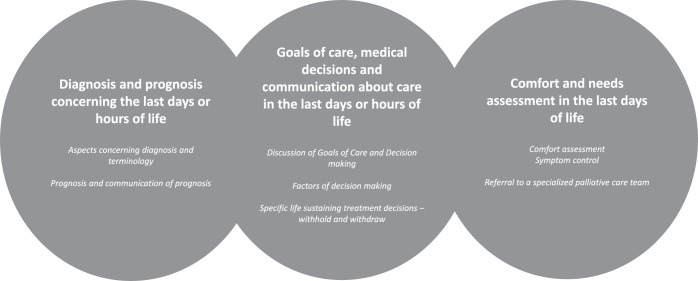
Themes and sub-themes which emerged from reflexive thematic analysis.

The MR reveal, in part, how physicians approach patients with dementia in the last days of life in the ED. Most relevant issues are presented under three main themes (Fig. 1). More details on coding are presented in Table 1.

**Table 1.** Codes, Themes and Subthemes

CODES	References (n = )	Themes	Subthemes
End-of-life Terminal situation Last days or hours of life Agony Advanced disease Critical state Bad prognosis Imminent failure Surrogate and family communication	105	Diagnosis and prognosis concerning the last days or hours of life	Aspects concerning Diagnosis and Terminology
			Prognosis and Communication of Prognosis
Age Comorbidities Performance status Senior discussion/Other Surrogate and family discussion Response to treatment Do not resuscitate decision Diagnosis withhold Treatment withdraw Treatment withhold Comfort care Conservative care	158	Goals of care, medical decisions and communication about care in the last days or hours of life	Discussion of Goals of Care and Decision making
			Factors of decision making
			Specific life sustaining treatment decisions – withhold and withdraw
Comfort measures Specialized palliative care Signs of physical discomfort	121	Comfort and needs assessment in the last days of life	Comfort assessment
			Referral to a specialized Palliative Care team

#### Diagnosis and prognosis concerning the last days or hours of life

##### Aspects concerning diagnosis and terminology

Identification of the clinical situation covered elements related to the acute clinical presentation (symptoms and signs) and the presence of advanced disease criteria. Mention to respiratory and circulatory symptoms and signs were preponderant,

*“respiratory and cardiovascular severe dysfunctions”* (MR 26)

and *“with Cheyne-Stokes respiratory pattern, interspersing periods of tachypnea with periods of apnea”* (MR 191).

*“Terminal cachexia. Rigid position characteristic of advanced dementia.”* (MR 108)

Despite a high Functional Assessment Staging Tool score this was rarely explicitly mentioned. Specific criteria of advanced dementia were only rarely indicated, while *“terminal heart failure”* (MR 146) and *“terminal renal failure”* (MR 4) represented some of the identified criteria of other associated advanced comorbidities.

Diagnosis concerning the critical state of the patient and its associate terminology were diverse. Terms like *“terminal”* and *“end of life”* were found in the majority of MR, while the specific designation of last days or hours of life were only twice mentioned as *“terminal patient, probable in the last hours of life”* (MR 200) and *“patient in an agonizing state”* (MR 30).

##### Prognosis and communication of prognosis

Prognostic registry disclosed the expected clinical deterioration and high risk of proximity to death. Most MR referred only *“prognosis”* integrated in the context of signs of clinical deterioration, reinforced by short expressions as *“bad prognosis”* (MR 69, MR 93, MR 118, MR 168, MR 190), *“bad evolution”* (MR 45, MR 113), *“reserved prognosis”* (MR 17, MR 30, MR 42, MR 94, MR 113, MR 124, MR 133, MR 135, MR 139, MR 150, MR 168, MR 172, MR 181, MR 186, MR 192, MR 193), *“probable fatal outcome”* (MR 53, MR 71, MR 93). Longer expressions were present, for example *“very reserved prognosis, with a high risk of unfavorable evolution and short-term cardiac arrest”* (MR 166).

Explicit prognosis communication to other members of the healthcare team was absent while communication to family and caregivers was usually present, in strait relation to prognosis. This communication addressed those who were contacted

*“the patient’s clinical situation and reserved prognosis were discussed with the family present (son and grandson) who understood”* (MR 165);

or attempted to be contacted *“an attempt was made to contact the daughter by telephone to inform her of the patient’s condition, without success”* (MR 158);

and often mentioned the degree of understanding by family member of the gravity of the clinical situation perceived by the clinician *“I communicated a very reserved prognosis to the family, who understood”* (MR 113);

and that family visits were facilitated *“I will contact the daughter, explain the cautious prognosis and plan a visit for this morning”* (MR 94).

#### Goals of care, medical decisions and communication about care in the last days or hours of life

##### Discussion of goals of care and decision making

References about goals of care were diverse. None directly mentioned the patients’ underlying values or priorities. Implicit information was present:

*“background data, functional status and decision on the last hospitalization already discussed with family/were considered”* (MR 154);

*“after discussing therapeutic objectives with the patient’s daughter, exclusive symptomatic treatment was decided”* (MR 40).

There was no information about previous advanced care planning. Decision making was frequently referred to as “team discussed,” in relation to discussion of goals of care and treatment decisions,

*“We decided as a team to prioritize the best supportive care”* (MR 158).

More specifically, peer and/or multidisciplinary discussions often included internal medicine senior physicians, *“After discussion with a senior physician, given the multiple advanced organic dysfunctions, we decided to suspend NIV”* (MR 125)

and intensive care colleagues *“Discussion with a colleague from the Intensive Care Unit, agreeing that, given the comorbidities of the patient with high functional fragility, there is no benefit on invasive support”* (MR 86).

The inclusion of Nursing professionals in these discussions was never explicit.

Different designations concerning goals of care of the last days or hours of life were present, namely: *“palliative care,” “comfort care,” “exclusively symptomatic treatment,” “supportive care”* and less frequent *“conservative treatment”* (MR 33, MR 34, MR 65, MR 74). These designations were frequently applied to decision-making, often followed by a lack of improvement with the treatment of potentially reversible causes. The attitudes they were associated with were essentially those of withdrawal or withholding. Designations were presented as equivalent terms and focused exclusively on symptom control.

##### Factors of decision making

Old age, global health status and performance measures were considered, *“Given that this is a patient with low functional reserve and a high degree of dependence, it was decided to prioritize comfort care”* (MR 200);

as well as potential benefit of the clinical intervention, like diagnostic testing, invasive measures and treatments, *“the benefit of being invasive, doing blood tests and medicating with antibiotics is very dubious”* (MR 54).

Also, reversibility or lack of clinical response to treatment of the cause of clinical deterioration was frequently mentioned, *“There was no improvement in the general condition [of the patient], with the measures instituted. Exclusively symptomatic treatment was decided”* (MR 7).

##### Specific life sustaining treatment decisions – Withhold and withdraw

Life sustaining measures were present regarding treatment limitation, *“no indication for escalating care”* (MR 10, MR 39, MR 111, MR 120) and the benefit of withholding or withdrawing specific measures. Diagnosis investigation, clinical procedures, invasive life support, resuscitation and use of some drugs were some of the mentioned measures. Most MR included *“do not resuscitate (DNR)”* and *“no indication for non-invasive ventilation”* (MR 107, MR 110, MR 155, MR 181), *“no indication for thrombolysis”* (MR 93), *“no indication for starting antibiotics”* (MR 67), *“haemodialysis discontinuation”* (MR 77) or *“non-invasive ventilation suspension”* (MR 86, MR 125).

#### Comfort and needs assessment in the last days of life

#### Comfort assessment

Comfort assessment was shortly recorded as the patient’s state, described as *“comfortable”* (MR 2, MR 15, MR 23, MR 48, MR 54, MR 56, MR 71, MR 75, MR 109, MR 110, MR 131, MR 177, MR 191), *“calm”* (MR 56, MR 75, MR 95), with *“no signs of discomfort”* (MR 97, MR 105), *“denies pain, dyspnoea or respiratory discomfort”* (MR 23) or, when with compromised comfort, as *“groans and localizes pain”* (MR 27), *“very complaining, uncomfortable groans”* (MR 66), *“PAINAD 8 = severe pain”* (MR 33) and *“polypneic with signs of suffering”* (MR 53). These descriptions were recorded sporadically by doctors at different times during the visit to the ED. References to comfort assessment rarely came up during the first observation, would sometimes come up before the application of symptom control measures and never systematically after their application.

##### Referral to a specialized palliative care team

Referral to a specialized palliative care team was found occasionally. Reasons recorded included the assessment of palliative care needs, support for decision making and, most frequently, support for care management, *“admitted for organizing care together with the palliative care team”* (MR 76, MR 116). Notes on the need for palliative care occurred during the initial assessment in the ED, or after an unfavourable evolution and/or multidisciplinary discussion. The outcome of the referral for PC was not specified in the MRs and no patient was assessed by a specialized team in the ED, or no notes were taken if that occurred.

## Discussion

### Key findings

Most of the patient’s MR (n = 146, 72,6%) had no explicit reference to the identification of the last days of life. This suggests either low recognition of this clinical situation, which might compromise prompt implementation of adequate quality care measures for end-of-life, or, reflects ED culture where everything is fast paced, including clinical notes taking, especially regarding end-of-life care, which calls for a more descriptive, and hence, longer text.

#### Diagnosis and prognosis concerning the last days or hours of life

Diagnoses relating to the patient’s critical condition were generally recorded, which probably indicated the doctor’s concern to do so. The terminology used to describe the associated prognosis were diverse. Terms such as *“terminal”* and *“end of life”* were frequently mentioned and seem to be used somehow as equivalents to the situation of the last days or hours of life. This inconsistency of terms is not desirable. An active attitude to diagnose and do so correctly is recommended (Díez-Manglano et al. 2021). The use of validated instruments for this purpose can be an added value (Cardona-Morrell and Hillman 2015; Walter et al. 2001).

In addition, the frequent use of verbal communication in the context of ED, as demonstrated by Currie et al (Currie et al. 2003), may contribute to and partly explain the limited recording of this situation. Prognostic registry disclosed the expected clinical deterioration. Contrary to recommendations (van der Steen et al. 2014), multidisciplinary clinical identification of the last days of life and its communication to other members of the healthcare team were not registered in the patients’ notes. However, communication to family and caregivers was usually present, in strait relation to prognosis.

#### Goals of care, medical decisions and communication about care in the last days or hours of life

Rather than the discussion of goals of care with the patient and the family, the analysis of the MR suggests that family communication focuses on communicating the prognosis and promoting their presence at this critical moment, which are only two of the important measures in end-of-life care (van der Steen et al. 2014). Patient information, including demographic, clinical and social data, was previously identified as the most needed information in the ED (Ayatollahi et al. 2013; Reddy and Spence 2008) and this is normally obtained by asking patients and/or their relatives (McKnight et al. 2001; Reddy and Spence 2008). Furthermore, even if there is information recorded in the MR regarding ACP and patient’s decisions, often those notes can be overlooked by other professionals, which may reflect the realities of data fragmentation in EDs and hospital wards. Information and registration breakdowns can increase the risk of communication failures with potential adverse effects on patients(Hertzum 2010).

And even when there has been some advanced discussion about patients’ decisions, it turns out that MR of the advanced or end-of-life stages of dementia can be overlooked by other professionals, which may reflect the fragmentation of data sharing in emergency departments and hospital wards.

Also, involvement of senior physicians in these discussions was recorded, however, that was not the case for nurses, as recommended in the literature (van der Steen et al. 2014).

Specific communication/discussion about goals of care and medical decisions with family members was absent in all MR. This is noteworthy because, not only, most of the patients didn’t present with an advanced care plan, but also, because the recommended model of shared-decision making implies active and ongoing discussion with surrogate and family decision makers about goals of care (McGlinchey et al. 2023; van der Steen et al. 2014). In fact, it is a process that should always be reviewed when there is a significant change in health status (McGlinchey et al. 2023; van der Steen et al. 2014). MR are likely to be of better quality if including information about preferences of end of life care (Sulmasy et al. 1996) since this can facilitate better access to the patient’s perspective, greater congruence in care and reduce the burden on the caregivers(Houben et al. 2014).

Our findings suggest that decision making notes reflect the global health status and performance of the patient as well as the potential benefit of the clinical intervention. The reversibility or lack of clinical response to treatment were also considered. These criteria are in line with the recommended focus being on the patient’s best interest achieved with a primary goal of maximization of comfort (van der Steen et al. 2014). Communication about PC and end-of-life care that can provide comfort care would reassure and comfort families, prevent their misunderstanding and guilty feeling of “giving up” on the patients.

Terms with probable equivalent meaning were present, e.g., *“palliative care,” “comfort care,” “exclusively symptomatic treatment,” “supportive care”* and *“conservative treatment.”* This is relevant because specific features of the ED care, such as task complexity, high speed healthcare delivery, frequent transfer of care between providers, multiple interruptions, high turnover of patients, and sometimes dealing with unknown or complex cases, makes this type of care prone to errors (Fattahi et al. 2023). Incorrect use of medical terms might increase this risk of mistakes. Use of standard terminology can improve clarity of record and eventually continuity of care. Also considering in the ED a specific set of essential outcomes for the best care of the dying person, as recently proposed by iLIVE(Zambrano et al. 2025), could improve care through standardization of clinical aspects of care, improving patient, family and healthcare professional’s experiences. This international Delphi study and consensus meeting composed a 14-item core outcome set: to address pain, to address anxiety, to address respiratory symptoms, to ensure that family and friends have unrestricted access to the patient, to address the fear of death, to give the possibility to say goodbye, to recognize and discuss the dying phase, to reduce suffering, to ensure dignity and respect, to ensure access to competent health professionals and to their continuous support, to provide compassionate care, to make the patient and the family feel heard and understood, to respect the patient’s autonomy, preferences and wishes and to provide quality of death and dying.

Withhold and withdrawal of life sustaining treatments were the main treatment decisions present in the MR. Recording these decisions seems useful and probably promotes better team communication and care since access to high quality data at the ED can be extremely important to improve quality and promptness of care (Ayatollahi et al. 2013; Hakimzada et al. 2008).

#### Comfort and needs assessment in the last days of life

Few references to the patient’s state of comfort were noted. Palliative care needs assessment and palliative care support for decision making were also only sporadically recorded. Emphasis on palliative care in the MR were almost always related to help support and organize further end-of-life care, and not so much in facilitating decision making or anticipating suffering, as proposed in the literature (McGlinchey et al. 2023; van der Steen et al. 2014). In fact, support for decision-making should focus on how the patient can be supported to make their own decisions and, if this is not possible, support families in their role as proxy decision-makers (Scholten and Gather 2018).

#### Study strengths and limitations

The main strength is that this is a real-world study based on ED MR. It includes clinical notes related to the identification of last days or hours of life, goals of care definition, communication and caring at the end-of-life of patients with dementia in the ED.

A limitation is that this is a single-centre study, but the biggest limitation is that the analysis is restricted to information recorded in the MR which reflects formally documented interactions and discussions. There is no possibility of knowing what else, if anything, was ever discussed and with whom. The MR does not fully represent actual practice. The limited time and facilitating conditions of registration in the ED can prevent the proper recording of clinical assessment, discussion and decision-making, as well as the application of palliative measures. This does not exclude that all the steps were not carried out, nor does recording them confirm that they were. Future research into the quality of care could involve complementing the analysis of the quality of the records with other indicators, both clinical and of the satisfaction of those involved, using qualitative research methods, such as interviews and focus groups. Ultimately it would be desirable to develop a systematic tool with common nomenclature to be used widely, and even internationally, with proper translation and validation.

## Final considerations

To our knowledge, this is the first qualitative study on end-of-life care provided in the ED to patients with dementia in the last days of life. Caring for patients with dementia at the end-of-life in the ED is challenging (Vieira Silva et al. 2025). Prompt and adequate clinical identification of the last days or hours of life is mandatory to provide the best care during this period (McGlinchey et al. 2023)^.^ MR should reflect this in the ED. Our analysis of the MR of the dying patients with dementia in the ED emerged with limited identification of this clinical situation and poor registration of aspects of communication and shared decision making. Just because this type of note taking is not prioritised, does not mean that conversations and discussions do not take place with all who should be involved in patient care. Family involvement and multidisciplinary team discussions are considered key measures and are in line with the patient’s best interest, especially when there is significant cognitive impairment (van der Steen et al. 2014). The dynamic nature of the ED care might benefit from more complete and accurate documentation of the care processes (Liaw et al. 2012). Additionally, appropriate use of medical terms and accurate record of goals of care and treatment might benefit end-of-life care. Consideration may be given to using a specific set of essential outcomes to better care for the dying person in the ED.

Further studies focusing on understanding which specific themes concerning best care in the last days or hours of life of patients with dementia, and how those should be recorded in the ED MR should be of great use to add to the evidence.

## References

[ref1] Anantapong K and Davies N (2021) Talking about future decision-making capacity and advance care planning in diagnosis disclosure of dementia. *International Psychogeriatrics* 33(11), 1119–1121. doi:10.1017/s1041610221000648 33926599

[ref2] Ayatollahi H, Bath PA and Goodacre S (2013) Information needs of clinicians and non-clinicians in the Emergency Department: a qualitative study. *Health Information & Libraries Journal.* 30(3), 191–200. doi:10.1111/hir.12019 23981020

[ref3] Braun V and Clarke V (2006) Using thematic analysis in psychology. *Qualitative Research in Psychology* 3(2), 77–101. doi:10.1191/1478088706qp063oa

[ref4] Braun V and Clarke V (2024) Supporting best practice in reflexive thematic analysis reporting in Palliative Medicine: a review of published research and introduction to the Reflexive Thematic Analysis Reporting Guidelines (RTARG). *Palliative Medicine* 38(6), 608–616. doi:10.1177/02692163241234800 38469804 PMC11157981

[ref5] Brinkman-Stoppelenburg A, Rietjens JA and van der Heide A (2014) The effects of advance care planning on end-of-life care: a systematic review. *Palliative Medicine* 28(8), 1000–1025. doi:10.1177/0269216314526272 24651708

[ref6] Cardona-Morrell M and Hillman K (2015) Development of a tool for defining and identifying the dying patient in hospital: criteria for Screening and Triaging to Appropriate aLternative care (CriSTAL). *BMJ Support Palliat Care* 5(1), 78–90. doi:10.1136/bmjspcare-2014-000770 PMC4345773 25613983

[ref7] Currie LM, Graham M, Allen M, et al. (2003) Clinical information needs in context: an observational study of clinicians while using a clinical information system. *AMIA Annual Symposium Proceedings* 2003, 190–194. 14728160 PMC1480255

[ref8] Detering KM, Hancock AD, Reade MC, et al. (2010) The impact of advance care planning on end of life care in elderly patients: randomised controlled trial. *British Medical Journal* 340, c1345. doi:10.1136/bmj.c1345 20332506 PMC2844949

[ref9] Díez-Manglano J, Sánchez Muñoz L, García Fenoll R, et al. (2021) Spanish and portuguese societies of internal medicine consensus guideline about best practice in end-of-life care. *Revista Clinica Espanola* 221(1), 33–44. doi:10.1016/j.rce.2020.04.014 33998477

[ref10] Donnelly S, Begley E and O’Brien M (2019) How are people with dementia involved in care-planning and decision-making? An Irish social work perspective. *Dementia (London)* 18(7-8), 2985–3003. doi:10.1177/1471301218763180 29544346

[ref11] Fattahi M, Keyvanshokooh E, Kannan D, et al. (2023) Resource planning strategies for healthcare systems during a pandemic. *European Journal of Operational Research* 304(1), 192–206. doi:10.1016/j.ejor.2022.01.023 35068665 PMC8759806

[ref12] Hakimzada AF, Green RA, Sayan OR, et al. (2008) The nature and occurrence of registration errors in the emergency department. *International Journal of Medical Informatics* 77(3), 169–175. doi:10.1016/j.ijmedinf.2007.04.011 17560165 PMC2259219

[ref13] Hegde S and Ellajosyula R (2016) Capacity issues and decision-making in dementia. *Annals of Indian Academy of Neurology.* 19(Suppl 1), S34–s39. doi:10.4103/0972-2327.192890 27891023 PMC5109759

[ref14] Hertzum M (2010) Breakdowns in collaborative information seeking: a study of the medication process. *Information Processing and Management* 46, 646–655. doi:10.1016/j.ipm.2009.10.005

[ref15] Houben CHM, Spruit MA, Groenen MTJ, et al. (2014) Efficacy of advance care planning: a systematic review and meta-analysis. *Journal of the American Medical Directors Association* 15(7), 477–489. doi:10.1016/j.jamda.2014.01.008 24598477

[ref16] Kantor MA, Scott BS, Abe-Jones Y, et al. (2021) Ask about what matters: an intervention to improve accessible advance care planning documentation. *Journal of Pain and Symptom Management* 62(5), 893–901. doi:10.1016/j.jpainsymman.2021.05.007 34000334

[ref17] Karnieli-Miller O, Werner P, Neufeld-Kroszynski G, et al. (2012) Are you talking to me?! An exploration of the triadic physician-patient-companion communication within memory clinics encounters. *Patient Education and Counseling* 88(3), 381–390. doi:10.1016/j.pec.2012.06.014 22789148

[ref18] Lafond P, Chalayer E, Roussier M, et al. (2016) A hospice and palliative care bed dedicated to patients admitted to the Emergency Department for End-of-Life Care. *American Journal of Hospice and Palliative Medicine* 33(4), 403–406. doi:10.1177/1049909114562947 25500432

[ref19] Liaw ST, Chen HY, Maneze D, et al. (2012) Health reform: is routinely collected electronic information fit for purpose? *Emergency Medicine Australasia* 24(1), 57–63. doi:10.1111/j.1742-6723.2011.01486.x 22313561

[ref20] Marck CH, Weil J, Lane H, et al. (2014) Care of the dying cancer patient in the emergency department: findings from a national survey of Australian emergency department clinicians. *Internal Medicine Journal* 44(4), 362–368. doi:10.1111/imj.12379 24528993

[ref21] Mashoufi M, Ayatollahi H and Khorasani-Zavareh AD (2019) Data quality assessment in emergency medical services: what are the stakeholders’ perspectives? *Perspectives in Health Information Management* 16, 1c. PMC6341415 30766454

[ref22] Mashoufi M, Ayatollahi H, Khorasani-Zavareh D, et al. (2023) Data quality assessment in emergency medical services: an objective approach. *BMC Emergency Medicine* 23(1), 10. doi:10.1186/s12873-023-00781-2 36717771 PMC9885566

[ref23] McGlinchey T, Early R, Mason S, et al. (2023) Updating international consensus on best practice in care of the dying: a Delphi study. *Palliative Medicine* 37(3), 329–342. doi:10.1177/02692163231152523 36734538 PMC10021119

[ref24] McKnight L, Stetson PD, Bakken S, et al. (2001) Perceived information needs and communication difficulties of inpatient physicians and nurses. *Proceedings of the AMIA (American Medical Informatics Association) Symposium* 453–457. PMC2243385 11825229

[ref25] Reddy MC and Spence PR (2008) Collaborative information seeking: a field study of a multidisciplinary patient care team. *Information Processing and Management* 44(1), 242–255. doi:10.1016/j.ipm.2006.12.003

[ref26] Scholten M and Gather J (2018) Adverse consequences of article 12 of the UN Convention on the Rights of Persons with Disabilities for persons with mental disabilities and an alternative way forward. *Journal of Medical Ethics* 44(4), 226–233. doi:10.1136/medethics-2017-104414 29070707 PMC5869457

[ref27] Sulmasy DP, Dwyer M and Marx E (1996) Do the ward notes reflect the quality of end-of-life care? *Journal of Medical Ethics* 22(6), 344–348. doi:10.1136/jme.22.6.344 8961119 PMC1377116

[ref28] Turley M, Wang S, Meng D, et al. (2016) Impact of a care directives activity tab in the electronic health record on documentation of advance care planning. *Perm J* 20(2), 43–48. doi:10.7812/tpp/15-103 27057820 PMC4867824

[ref29] van der Steen JT, Radbruch L, Hertogh CM, et al. (2014) White paper defining optimal palliative care in older people with dementia: a Delphi study and recommendations from the European Association for Palliative Care. *Palliative Medicine* 28(3), 197–209. doi:10.1177/0269216313493685 23828874

[ref30] Vieira Silva S, Conceição P, Antunes B, et al. (2025) Emergency department use and responsiveness to the palliative care needs of patients with dementia at the end of life: a scoping review. *Palliative and Supportive Care* 23, e51. doi:10.1017/S1478951524001627 39865850 PMC13166383

[ref31] Walter LC, Brand RJ, Counsell SR, et al. (2001) Development and validation of a prognostic index for 1-year mortality in older adults after hospitalization. *Jama* 285(23), 2987–2994. doi:10.1001/jama.285.23.2987 11410097

[ref32] Yash Pal R, Kuan WS, Koh Y, et al. (2017) Death among elderly patients in the emergency department: a needs assessment for end-of-life care. *Singapore Medical Journal* 58(3), 129–133. doi:10.11622/smedj.2016179 27917433 PMC5360867

[ref33] Yates J, Stanyon M, Samra R, et al. (2021) Challenges in disclosing and receiving a diagnosis of dementia: a systematic review of practice from the perspectives of people with dementia, carers, and healthcare professionals. *International Psychogeriatrics* 33(11), 1161–1192. doi:10.1017/s1041610221000119 33726880

[ref34] Zambrano SC, Egloff M, Gonzalez-Jaramillo V, et al. (2025) A core outcome set for best care for the dying person: results of an international Delphi study and consensus meeting. *Palliative Medicine* 39(1), 163–175. doi:10.1177/02692163241300867 39629728 PMC11673312

